# Bilateral Prostate Artery Embolization for Massive Benign Prostatic Hyperplasia in a Frail Elderly Patient With Recurrent Urinary Retention

**DOI:** 10.7759/cureus.109998

**Published:** 2026-05-31

**Authors:** Ana B Cuni Hernandez, Paige Carruthers, Elizabeth Blanco Espinosa, Idania Cruzata Matos, Claudia A Garcia Gonzalez, Laura Diaz Perez, Raysa Garces Ruiz

**Affiliations:** 1 Family Medicine, Atlas Urology, Bradenton, USA; 2 Urology, Atlas Urology, Bradenton, USA; 3 General Surgery, Hospital Arnaldo Milian, Villa Clara, CUB; 4 General Practice, Ceda Orthopedic Group, Miami, USA; 5 Neurosurgery, Hospital Arnaldo Milian, Villa Clara, CUB; 6 General Medicine, HCA Healthcare, Henderson, USA; 7 Critical Care, Faustino Perez Hospital, Matanzas, CUB; 8 Family Medicine, Included Health, Lewiston, USA; 9 Family Medicine, Institute of Medical Sciences of Camaguey, Camaguey, CUB

**Keywords:** bph, interventional radiology, lower urinary tract symptoms, prostate artery embolization (pae), urinary retention (ur)

## Abstract

Benign prostatic hyperplasia (BPH) is a highly prevalent condition among elderly men and a major cause of lower urinary tract symptoms (LUTS), urinary retention, recurrent urinary tract infections (UTIs), hematuria, and bladder outlet obstruction (BOO). Management of severe BPH in elderly patients with multiple comorbidities remains challenging, particularly in those who are poor surgical candidates. Prostate artery embolization (PAE) has emerged as a minimally invasive alternative for symptomatic relief in patients with moderate-to-severe LUTS secondary to BPH. We present the case of an 87-year-old male with massive BPH (estimated prostate volume 263 g) complicated by recurrent urinary retention, recurrent catheter-associated UTIs (CAUTIs), gross hematuria, and chronic Foley catheter dependence despite maximal medical therapy with tamsulosin and finasteride. His medical history was significant for coronary artery disease with prior cardiac stent placement, prior stroke, chronic anticoagulation therapy, hypertension, nephrolithiasis, and cognitive impairment, making him a high-risk candidate for conventional surgical intervention. Imaging and cystoscopic evaluation confirmed severe prostatomegaly with intravesical prostatic protrusion and BOO. The patient underwent successful bilateral PAE via left radial artery access using 300-500 μm embospheres without procedural complications. At follow-up, he demonstrated marked symptomatic improvement with restoration of spontaneous voiding, absence of recurrent urinary retention, and reduction of post-void residual volume to 35 mL. No further catheter obstruction or catheter-associated infections were reported. This case highlights the effectiveness and safety of PAE as a minimally invasive therapeutic option for elderly high-risk patients with giant BPH and chronic catheter dependence. The favorable clinical outcome observed further supports the growing role of PAE in complex geriatric urologic populations who are not ideal candidates for traditional surgical management.

## Introduction

Benign prostatic hyperplasia (BPH) is one of the most common age-related diseases affecting men worldwide and represents a major cause of lower urinary tract symptoms (LUTS) in the aging population. Epidemiological studies estimate that histologic evidence of BPH is present in approximately 50-60% of men in their sixth decade of life and increases to nearly 80-90% among men older than 80 years [1-3]. The prevalence of moderate-to-severe LUTS secondary to BPH continues to rise globally as life expectancy increases and the elderly population expands [3]. 

BPH results from progressive stromal and epithelial hyperplasia within the transition zone of the prostate, leading to prostate enlargement and bladder outlet obstruction (BOO). Clinical manifestations include urinary frequency, urgency, nocturia, hesitancy, intermittency, weak urinary stream, incomplete bladder emptying, and urinary retention [1]. Progressive disease may result in significant complications, including recurrent urinary tract infections (UTIs), gross hematuria, bladder stones, hydronephrosis, renal dysfunction, and chronic catheter dependence [1,4]. Acute urinary retention remains one of the most serious complications of BPH and is associated with substantial morbidity, recurrent hospitalizations, and reduced quality of life in elderly patients [4]. 

The healthcare burden associated with BPH is considerable. BPH-related LUTS account for millions of outpatient visits annually and remain among the leading causes of urologic intervention in older men. Chronic Foley catheterization in patients with refractory urinary retention further increases morbidity due to recurrent catheter-associated UTIs (CAUTIs), bladder spasms, hematuria, delirium, and decreased functional status [5]. Elderly patients with chronic catheter dependence are particularly vulnerable to recurrent infections and repeated hospital admissions, contributing significantly to healthcare utilization and costs [5]. 

Medical therapy with alpha-adrenergic antagonists and five-alpha reductase inhibitors remains the standard first-line treatment for symptomatic BPH. However, patients with markedly enlarged prostates frequently experience progression despite pharmacologic management and may ultimately require procedural intervention [1]. Transurethral resection of the prostate (TURP) has historically been considered the gold standard surgical treatment for moderate-to-severe LUTS secondary to BPH. Nevertheless, elderly patients with advanced cardiovascular disease, chronic anticoagulation, prior stroke, or frailty may not tolerate surgical intervention because of increased anesthetic and perioperative risks [6]. 

Over the past decade, prostate artery embolization (PAE) has emerged as a promising minimally invasive alternative for selected patients with symptomatic BPH. The procedure involves selective embolization of the prostatic arterial supply, resulting in ischemic infarction and subsequent reduction in prostate volume, thereby improving BOO and urinary symptoms [2,7]. Recent evidence has demonstrated significant improvements in the International Prostate Symptom Score (IPSS), quality of life, urinary flow rates, and post-void residual urine volume following PAE while maintaining a favorable safety profile [2,7]. Importantly, updated American Urological Association (AUA) guidelines published in 2023 formally incorporated PAE as a treatment option for appropriately selected patients with LUTS secondary to BPH, reflecting the growing evidence supporting its efficacy and safety [1,2].

Several recent studies suggest that PAE may be particularly advantageous in elderly patients with giant prostates and significant medical comorbidities, especially those considered poor candidates for conventional surgery [2,7]. Additionally, successful restoration of spontaneous voiding following PAE has been reported in patients with chronic urinary retention and long-term catheter dependence, further expanding its role in complex geriatric urology [7]. 

Despite growing evidence supporting PAE for symptomatic BPH, reports involving medically complex geriatric patients with giant prostate enlargement, chronic catheter dependence, and significant surgical risk remain relatively limited. Documenting such cases contributes to the expanding evidence supporting minimally invasive management strategies and further highlights the evolving role of PAE in elderly patients who are poor candidates for conventional surgical intervention.

## Case presentation

An 87-year-old Caucasian male with a past medical history significant for BPH, recurrent urinary retention, UTIs, nephrolithiasis, hypertension, coronary artery disease status post-cardiac stent placement, atrial fibrillation on anticoagulation, prior stroke, and memory impairment presented for urologic follow-up with an approximately four-year history of progressively worsening LUTS and recurrent urinary retention despite medical therapy.

The patient initially established urologic care in December 2024 for management of longstanding BPH with urinary retention. At that time, symptoms were reportedly well controlled with tamsulosin therapy, and he denied significant voiding difficulties. However, over the following months, he experienced progressive obstructive urinary symptoms characterized by urinary frequency, nocturia up to four times, weak urinary stream, straining, incomplete bladder emptying, and intermittent episodes of acute urinary retention.

In November 2025, the patient presented to the emergency department with acute urinary retention requiring a Foley catheter placement. Following catheterization, gross hematuria developed without clot formation. He was subsequently managed with continuation of tamsulosin, initiation of finasteride, and antibiotic therapy. Despite an initially successful voiding trial, the patient continued to experience recurrent episodes of urinary retention and recurrent UTIs requiring repeated catheter exchanges approximately every two weeks.

**Figure 1:**
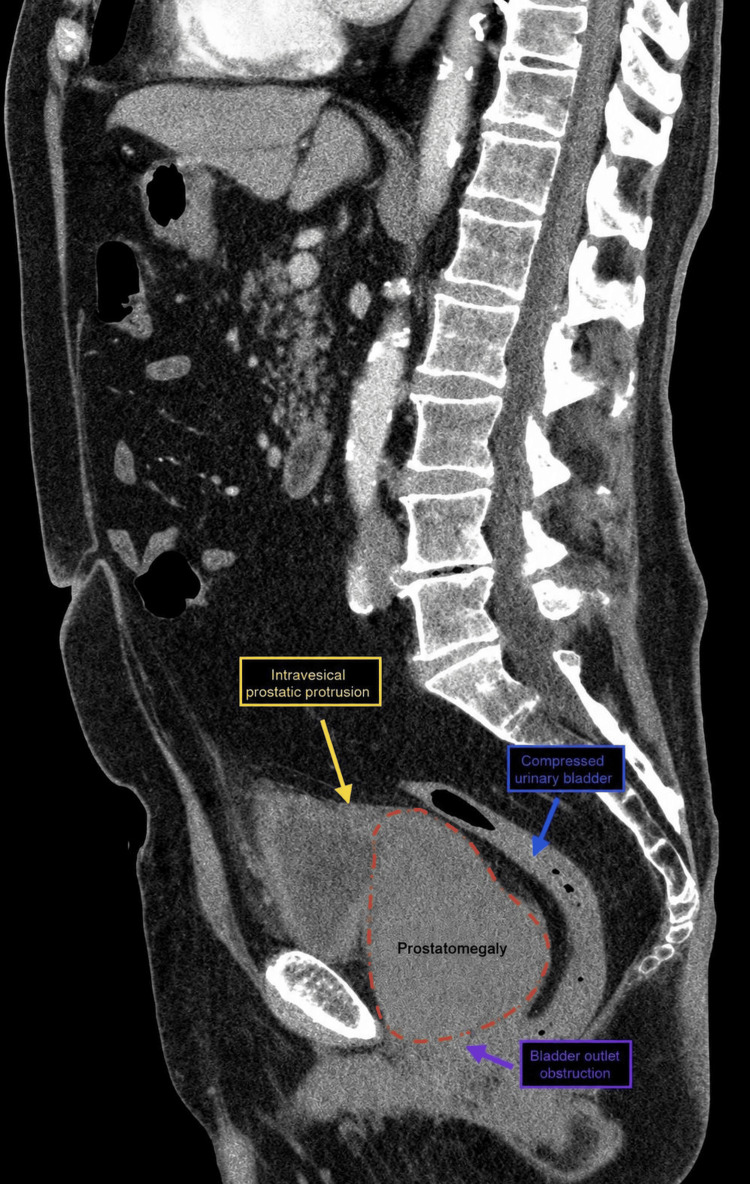
Sagittal contrast-enhanced CT Image demonstrating marked prostatomegaly with significant intravesical prostatic protrusion causing bladder outlet obstruction

**Figure 2:**
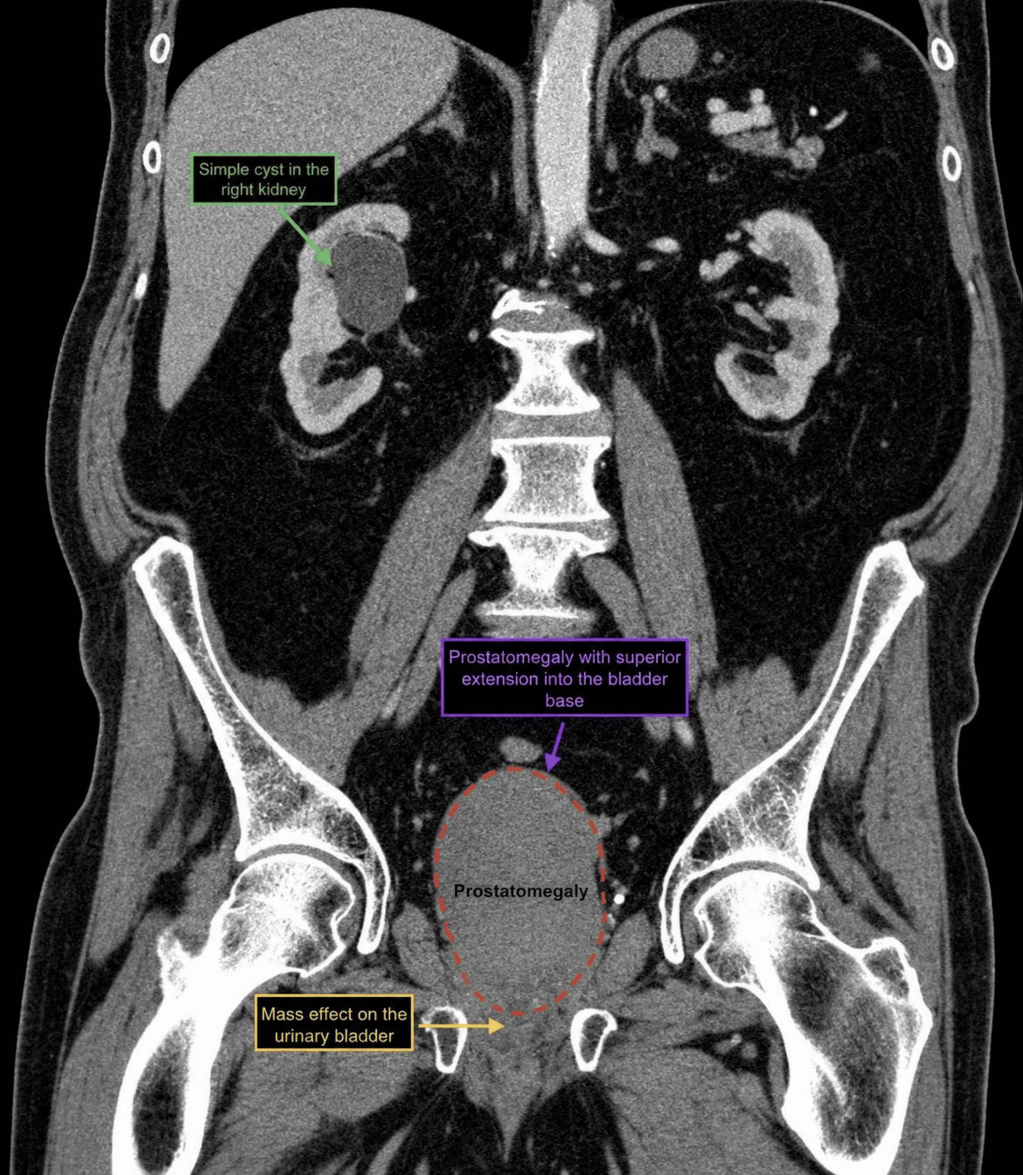
Coronal contrast-enhanced CT Image showing massive enlargement of the prostate gland with mass effect on the urinary bladder. Simple cyst in the right kidney.

**Figure 3:**
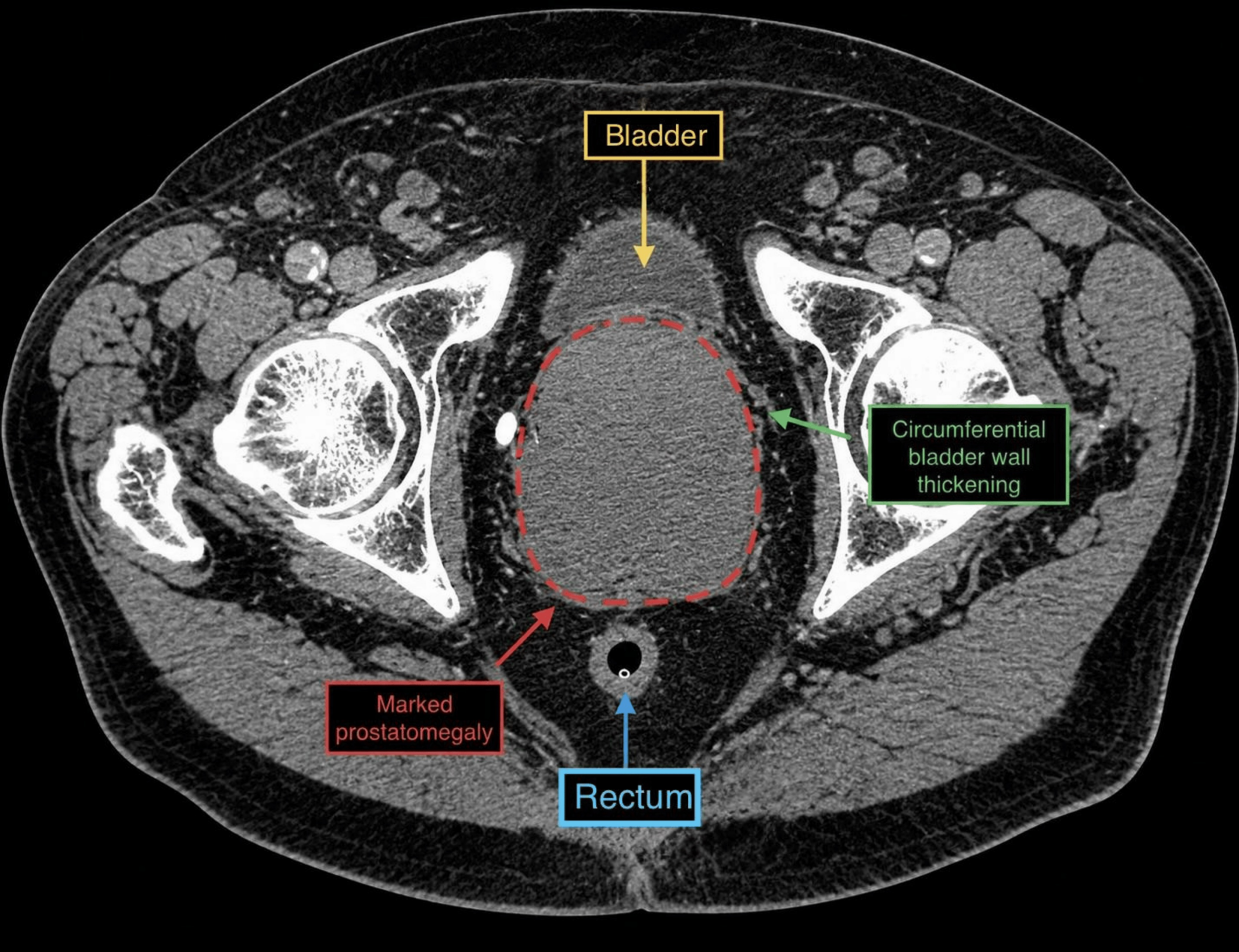
Axial contrast-enhanced CT Image demonstrating enlarged prostate and circumferential bladder wall thickening.

Computed tomography urography (CTU) performed in December 2025 demonstrated marked prostatomegaly measuring 6.7 cm with intravesical prostatic protrusion causing possible BOO. Circumferential bladder wall thickening measuring 1.2 cm suggested chronic cystitis or BOO. Additional findings included a non-obstructive 7.5 mm right renal calculus, a 4.8 cm simple right renal cyst, a small hiatal hernia, bilateral fat-containing inguinal hernias, and chronic calcified granulomatous changes involving the liver and spleen. The prostate volume was estimated at approximately 263 g (Figures 1-3).

Flexible cystoscopy performed in January 2026 revealed a massively enlarged prostate and diffuse bladder edema attributed to chronic catheterization. The anterior and posterior urethras were otherwise unremarkable. Both ureteral orifices were patent with clear urine efflux, and no bladder tumors or intravesical abnormalities were identified. Bladder mucosa appeared otherwise normal. Urinary cytology and fluorescence in situ hybridization (FISH) specimens were obtained (Table 1). At that time, a physical examination revealed an elderly male in no acute distress with a soft, non-distended abdomen without suprapubic tenderness, palpable bladder distension, or costovertebral angle tenderness. The patient’s urinary symptoms had become increasingly burdensome despite maximal medical therapy with an alpha-blocker and five-alpha reductase inhibitor treatment.

**Table 1:** Urinary malignancy evaluation with cytology and UroVysion fluorescence in situ hybridization (FISH) UroVysion® FISH assay (Abbott Molecular, Abbott Laboratories, Des Plaines, Illinois, USA)

Test	Patient	Result Reference	Interpretation
Urine Cytology	Negative for high-grade urothelial carcinoma	Negative for malignant urothelial cells	No cytologic evidence of high-grade urothelial malignancy
UroVysion FISH Assay	Negative	Negative	No chromosomal abnormalities were detected associated with urothelial carcinoma

Given the patient’s advanced age, significant cardiovascular comorbidities, chronic anticoagulation use, and absence of clinical suspicion for prostate malignancy, prostate-specific antigen (PSA) evaluation was not obtained as part of the preprocedural workup and therefore remained unknown. Formal uroflowmetry and urodynamic studies were not performed, as treatment decisions were guided by the overall clinical presentation, imaging findings, cystoscopic evaluation, and recurrent catheter-dependent urinary retention.

Differential diagnostic considerations included BOO secondary to severe BPH, chronic cystitis, and impaired bladder emptying related to advanced age and prior cerebrovascular disease. However, the presence of marked prostatomegaly with intravesical protrusion, recurrent urinary retention, cystoscopic evidence of massive prostatic enlargement, and persistent symptoms despite medical therapy supported clinically significant BOO as the predominant etiology.

Preprocedural assessment demonstrated severe LUTS with an IPSS [8] of 23 and a quality-of-life score of 5, reflecting substantial symptom burden. Considering the patient’s recurrent urinary retention and poor candidacy for major surgical intervention, minimally invasive management with PAE was pursued.

The patient subsequently underwent successful bilateral PAE in March 2026 via left radial artery access under moderate conscious sedation. Embolization of both prostatic arteries was performed using 300-500 µm embospheres until near stasis was achieved bilaterally. Completion angiography demonstrated successful embolization without evidence of immediate complications. Hemostasis was achieved with a radial compression band, and the patient tolerated the procedure well. Postprocedural management included ciprofloxacin, anti-inflammatory therapy, continuation of alpha-blocker therapy, bladder spasm prophylaxis, and analgesic medications.

Follow-up evaluation approximately four weeks after embolization demonstrated marked symptomatic improvement. The patient and his wife reported substantial improvement in urinary symptoms and overall quality of life. Postprocedural symptom assessment demonstrated marked improvement, with IPSS decreasing from 23 preprocedurally to 2, and quality-of-life score improving from 5 to 0 (“delighted”). The patient was voiding spontaneously without catheter dependence, and no additional episodes of urinary retention were reported. Post-void residual volume measured was 35 mL during follow-up assessment. The patient remained clinically stable on tamsulosin and finasteride therapy, and no postprocedural complications were reported.

## Discussion

This case highlights the significant clinical challenges associated with severe BPH in elderly patients with multiple comorbidities. It demonstrates the growing importance of PAE as a minimally invasive treatment option for high-risk surgical candidates [2,4]. 

Our patient presented with progressive LUTS complicated by recurrent urinary retention, recurrent CAUTIs, gross hematuria, and chronic Foley catheter dependence despite combination medical therapy with tamsulosin and finasteride. Imaging revealed severe prostatomegaly with an estimated prostate volume of approximately 263 g and significant intravesical prostatic protrusion causing BOO. Giant BPH of this magnitude is associated with increased risk of chronic bladder dysfunction, recurrent urinary retention, hydronephrosis, and recurrent infections due to longstanding outlet obstruction [4]. 

An important aspect of this case is the morbidity associated with chronic Foley catheterization. Long-term indwelling catheters are strongly associated with recurrent CAUTIs, bladder inflammation, obstruction, urethral trauma, hematuria, and recurrent hospital admissions, particularly in elderly patients [5]. Chronic catheter dependence may also negatively affect quality of life and contribute to progressive functional decline and delirium in frail geriatric populations [5]. In our patient, catheter exchanges were required every two weeks due to recurrent obstruction and infection, illustrating the substantial burden associated with chronic urinary retention. 

Conventional surgical management of giant BPH includes TURP, holmium laser enucleation of the prostate (HoLEP), and simple prostatectomy. However, these interventions may be associated with prolonged operative times, increased bleeding risk, transfusion requirements, and higher perioperative morbidity in elderly patients with cardiovascular disease and chronic anticoagulation therapy [6]. Our patient’s advanced age, prior stroke, coronary artery disease with cardiac stent placement, chronic antiplatelet therapy, and cognitive impairment significantly increased his surgical risk and made minimally invasive treatment particularly desirable. 

PAE offered several advantages in this setting. The procedure can be performed under local anesthesia with conscious sedation, thereby minimizing cardiopulmonary stress and reducing perioperative complications [2]. Additionally, embolization carries a lower bleeding risk than conventional surgery, which is especially important in patients requiring ongoing anticoagulation or antiplatelet therapy [2,6]. In this case, successful bilateral embolization was achieved via transradial access without procedural complications. 

The favorable outcome observed in this patient is consistent with recent literature evaluating the efficacy of PAE in severe BPH. Contemporary systematic reviews have demonstrated significant improvements in IPSS, quality of life, urinary flow parameters, and prostate volume reduction following embolization [7]. Furthermore, recent evidence suggests that larger prostates may undergo substantial volumetric reduction after embolization, supporting the role of PAE in giant BPH, in which surgical management becomes technically challenging [9]. 

Another particularly important finding in this case was the successful restoration of spontaneous voiding after prolonged Foley catheter dependence. Chronic urinary retention may lead to progressive detrusor dysfunction, raising concern regarding the ability to recover bladder contractility after relief of obstruction. Despite these concerns, our patient achieved catheter independence with a post-void residual volume of 35 mL following PAE. This finding supports growing evidence that PAE can facilitate catheter removal and restore spontaneous voiding in selected patients with severe BPH and chronic retention [7]. 

Recent guideline updates further strengthen the clinical relevance of PAE. The 2023 amendment to the AUA guidelines formally recognized PAE as a therapeutic option for LUTS secondary to BPH in appropriately selected patients, reflecting increasing evidence supporting its efficacy and safety profile [1,2]. This represents a major advancement in the multidisciplinary management of BPH and highlights the expanding collaboration between urology and interventional radiology. 

Despite promising results, several limitations of PAE should be acknowledged. Clinical improvement may occur more gradually compared with TURP, and some patients may require repeat embolization or additional procedures over time [7]. Moreover, successful embolization depends heavily on operator expertise and a detailed understanding of variable pelvic arterial anatomy. Although uncommon, non-target embolization remains a potential complication [2]. Nevertheless, current evidence supports the favorable safety profile of PAE, particularly in elderly patients with substantial surgical risk [2,7]. 

This case further contributes to the growing body of evidence supporting PAE as an effective and safe minimally invasive treatment modality for elderly patients with giant BPH, recurrent urinary retention, recurrent catheter-associated infections, and chronic Foley catheter dependence who are poor candidates for conventional surgery. 

The limitations of this report should be acknowledged in the context of the available clinical and follow-up data. PSA evaluation was not obtained as part of the preprocedural or follow-up assessment, given the absence of clinical suspicion for prostate malignancy and the management focus on severe symptomatic BOO. Formal uroflowmetry and urodynamic studies were also not performed, as clinical decision-making was guided by the patient’s overall presentation, imaging findings, cystoscopic evaluation, and recurrent catheter-dependent urinary retention. Additionally, repeat prostate volume assessment was not available during the short follow-up interval, as volumetric reduction following PAE typically evolves gradually and is more commonly evaluated over longer periods, often three to six months after embolization. Despite these limitations, clinically meaningful early improvement was documented through restoration of spontaneous voiding, reduction in post-void residual volume, catheter independence, and marked improvement in IPSS and quality-of-life scores.

## Conclusions

This case highlights the ability of PAE to achieve meaningful early clinical recovery in an elderly patient with massive BPH, recurrent urinary retention, and chronic Foley catheter dependence. Rapid restoration of spontaneous voiding, reduction in catheter-related burden, and favorable short-term outcomes following embolization support PAE as a valuable minimally invasive therapeutic option in medically complex patients who may not tolerate traditional surgery. As experience with PAE continues to expand, early functional outcomes such as those observed in this case may hold particular clinical significance in frail geriatric populations.
